# Neural correlates of affective theory of mind in medication-free nonsuicidal self-injury: An fMRI study

**DOI:** 10.3389/fpsyt.2022.850794

**Published:** 2022-07-22

**Authors:** Hyeri Moon, Gieun Nam, Ji-Won Hur

**Affiliations:** ^1^School of Psychology, Korea University, Seoul, South Korea; ^2^Department of Psychology, Chung-Ang University, Seoul, South Korea

**Keywords:** nonsuicidal self-injury, affective ToM, theory of mind, fMRI, Reading the Mind in the Eyes Test

## Abstract

Emerging evidence indicates that emotion processing deficits are associated with nonsuicidal self-injury (NSSI). However, limited attention has been paid to the socio-affective functions of NSSI. In this study, we aimed to investigate the affective theory of mind (ToM) in medication-free individuals engaging in NSSI at both behavioral and neural levels. Twenty-eight individuals (mean age = 22.96 years) who engaged in NSSI and 38 age-, sex-, and IQ-matched controls (mean age = 22.79 years) underwent functional magnetic resonance imaging while performing the “Reading the Mind in the Eyes Test” (RMET). All participants also completed the Difficulties in Emotion Regulation Scale (DERS), Toronto Alexithymia Scale (TAS-20), and Beck Scale for Suicide Ideation (BSI). Although we did not find significant group differences in the RMET performance, the NSSI group, relative to the controls, exhibited significantly greater left medial superior frontal lobe activation and decreased right angular gyrus activation than did the control group. Reduced right angular gyrus activity was related to higher DERS and TAS scores across all participants. Our findings provide new evidence for aberrant neural processing of affective ToM in self-injurers. Future studies in developing intervention protocols for NSSI should focus on the multifaceted phases of socio-affective processing.

## Introduction

Nonsuicidal self-injury (NSSI) is defined as the deliberate damage to one’s body tissue without conscious suicidal intent (1). Currently, NSSI has been deemed as a considerable public health burden, especially because of its close relationship with the transition to suicide (2, 3). Moreover, its prevalence has been increasing in the developed world (4). Considering that the prevalence of sleep disorders, depression, and anxiety, which are risk factors for NSSI, increased during the coronavirus disease 2019 pandemic, it is expected that the incidence of NSSI will increase in the long term (5–7).

The most recent Diagnostic and Statistical Manual of Mental Disorders (8) identified NSSI, which was considered to be one of the symptoms of mental illnesses such as borderline personality disorder, as “a condition in need of further study.” Although NSSI is not an official mental disorder diagnosis yet, the research on the underlying pathophysiology of NSSI is increasing at a relatively fast pace since it has a severe impact on public mental health. Previous research has suggested that abnormal emotional processing plays a significant role in the onset and maintenance of NSSI (9, 10).

More specifically, there is evidence of a relationship between NSSI and emotional processing deficits such as facial emotion recognition difficulties (11), alexithymia (12, 13), and emotion regulation problems (14–17). Indeed, the individuals who engaged in NSSI, compared to controls, are also more likely to experience consistent negative valence emotions (18) and report difficulties regulating their negative emotions (19, 20). In addition, a recent experimental study also showed a significant relationship between interpersonally focused negative and acute NSSI behaviors (21).

Of note, interpersonal vulnerabilities have also been suggested as the core psychopathology for the development and maintenance of NSSI in Nock’s hypothetical model (22). However, despite the strong assumption that NSSI is closely related to individuals’ social functions (23, 24), little is known about the socio-affective functions of those with NSSI (25). According to studies to date, people with NSSI have interpersonal disturbances, which may be the result of a biased perception of social-affective stimuli (26). Laghi et al. (27) have shown the poor performance in the Reading the Mind in the Eyes Test (RMET) of adolescents with NSSI compared to controls (mean age = 15; only girls; NSSI, 24.8 ± 4.2 vs. controls, 28.7 ± 3.9; Mann–Whitney *U* test total score = 57.5) in their pilot experimental study which aimed to verify potential difficulties in facial emotion discrimination in adolescents with NSSI diagnosis.

The RMET was originally designed to assess the theory of mind (ToM) deficits in high-functioning autism by Baron-Cohen et al. (28). ToM, a core aspect of social cognition in human beings (29), is the higher-order ability to attribute mental states to others, such as beliefs, feelings, intentions, or desires (30), and comprises two dimensions: affective ToM and cognitive ToM. Affective and cognitive ToM are closely related and share several brain regions (31); however, these are distinctive in that affective ToM refers to the capacity to infer the mental and emotional states of others, whereas cognitive ToM refers to the ability to infer the beliefs and intentions of others (32).

In the RMET, participants are asked to select one adjective out of four alternative labels that best describe the mental state depicted in an eye region photograph. The successful completion of the RMET requires the ability to infer and attribute others’ complex emotional mental states rather than simply label emotions (33). Given that looking into the eyes of others is key to developing and utilizing an affective ToM (34) and that many functional magnetic resonance imaging (fMRI) studies have determined social cognitive networks related to emotion decoding during RMET performance (35, 36), the RMET is now widely used to probe affective ToM (37).

Previous functional neuroimaging studies have found that affective ToM recruits broad brain areas, including the bilateral medial prefrontal cortex, temporoparietal junction (TPJ), and middle temporal gyrus (38–40). A neuroimaging meta-analysis examining 144 independent studies (39) indicated that affective ToM elicited greater activation in the posterior medial frontal cortex, bilateral inferior frontal gyrus, middle frontal gyrus, temporal poles, and posterior middle temporal gyrus, whereas cognitive ToM leads to increased activation in the precuneus, bilateral temporoparietal function, and right middle temporal gyrus. Moreover, affective ToM processing, relative to cognitive ToM, might be sensitive to pathological disturbances because the former is a more complex social cognition that relies on a deeper and broader network than the latter (32, 39, 41). Thus, it seems promising that using affective ToM tasks can be helpful for the understanding of the socio-affective characteristics of those with NSSI (27). However, to date, except for the previously mentioned behavioral pilot study, no neuroimaging study about affective ToM in NSSI has been conducted.

In summary, we aimed to identify neural mechanisms underlying affective ToM in NSSI. We hypothesized that individuals who cope with distress by engaging in repetitive self-injury might exhibit altered neural activity in response to the affective ToM task adopted from the RMET. Given the advantages of using neuroimaging research (42), we expected to clarify functional alterations in affective ToM in the NSSI group more closely than a behavioral task. Based on the Research Domain Criteria perspectives, we focused on the neurobiological anomalies in “social brain” network, including the medial prefrontal cortex and TPJ that could be a potential biomarker of NSSI (25, 43). It was also expected that greater alexithymia and emotion regulation problems— the general indicators of emotion processing deficits— would be associated with the aberrant brain function in NSSI.

## Materials and methods

### Participants

Twenty-eight medication-free participants (21 women) engaging in NSSI and 38 age-, sex-, and IQ-matched controls (26 women) without current illness were recruited through word-of-mouth and advertising on social media pages. The 28 participants enrolled in the NSSI group had engaged in five or more NSSI episodes in the past year. The Structured Clinical Interview for DSM-5 Disorders (SCID-5) (44) was administered to identify their psychiatric comorbidities other than NSSI. Thirty-eight controls also completed the Structured Clinical Interview for Non-patients (SCID-I/NP) (45). The handedness of all participants was assessed using the Edinburgh Handedness Inventory (46).

The exclusion criteria were as follows: (1) lifetime occurrence of psychotic symptoms or neurological disorder history; (2) current psychiatric treatment; (3) medication use in the previous month; (4) estimated IQ on the short form of the Korean version of the Wechsler Adult Intelligence Scale, Fourth Edition (47) < 80 (the lower limit of a normal IQ); (5) age < 19 or > 30; and (6) a familial history of psychosis. There were no intergroup differences in age, sex, and estimated IQ; therefore, no covariates were included in the statistical analyses. All participants completed the experiment after passing a magnetic resonance imaging safety screen (e.g., claustrophobia or ferrous metals in the body) and provided written informed consent reviewed by the university institutional review board in accordance with the Declaration of Helsinki.

### Measures

#### Toronto alexithymia scale (TAS-20)

A tool used to assess alexithymia, a subclinical condition characterized by a lack of emotional awareness (48). A higher score indicated a higher tendency for alexithymia. The TAS-20 considers three factors: difficulty identifying feelings, difficulty describing feelings, and externally oriented thinking. Each item is scored on a five-point Likert scale, and Cronbach’s alpha of the Korean version of the TAS (TAS-20) was 0.76 (49).

#### Difficulties in emotion regulation scale (DERS)

The DERS is a 36-item self-report measure that assesses the properties of emotion dysregulation (50). The Korean version of the DERS (51) used in this study is similar to the original scale which includes six factors: non-acceptance of emotional responses, difficulties engaging in goal-directed behavior, impulse control difficulties, lack of emotional awareness, limited access to emotion regulation strategies, and lack of emotional clarity. The Korean version also includes six factors: non-acceptance of emotions, difficulties in engaging in goal-directed behavior, impulse control difficulties, lack of attention to and awareness of emotions, limited access to emotion regulation strategies, and lack of emotional clarity. Total DERS scores were used in this study. Each item is scored as 1 = barely, or 5 = almost. Cronbach’s alpha for the DERS was 0.93 (51).

#### Beck scale for suicide ideation (BSI)

The BSI is a 19-item self-report measure consisting of statements that describe the presence and intensity of a participant’s ideation, plans, and intent to commit suicide. Each item is rated on a three-point Likert scale ranging from zero to two. The BSI includes three factors: (1) desire for death, (2) preparation for death, and (3) active suicidal desire. A high total score indicates higher levels of suicidal thoughts, and Cronbach’s alpha of the Korean version of the BSI was 0.74 (52).

### Scanning protocols and image acquisition

All structural and functional images were acquired using a 3.0-T Siemens Trio MRI scanner (Siemens Healthcare, Germany) at the Seoul National University Brain Imaging Center. All images were acquired using a 32-channel head coil. A series of 37 slices parallel to the anterior-posterior commissure plane, with a thickness of 3.4 mm, were collected using a T2*-weighted spin echo-planar image (6) sequence [repetition time (TR) = 2,200 ms, echo time (TE) = 30 ms, matrix = 2.5 × 2.5 × 3.4 mm^3^, field of view (FoV) = 210 mm, and flip angle = 90°]. T1-weighted multiband high-resolution images were also acquired for structural analysis with the following sequence: [TR = 2,400 ms, TE = 2.19 ms, matrix = 0.8 mm isotropic voxel, FoV = 272 mm, and flip angle 8°].

### Procedure and task

#### Task procedure

Written informed consent was collected from the participants. Then, participants completed MRI scans and self-reported questionnaires. In this procedure, one participants’ questionnaire was missing.

#### fMRI task design

The RMET (53) was employed for the affective ToM and control conditions. Every stimulus, which consisted of the eye region of a face in a photograph, was accompanied by four words (three distractors, one target), describing distinguishable emotional (affective ToM; i.e., RMET condition) or physical status (i.e., control condition). The three distractor words and one target word were positioned at the four corners of each stimulus. In the RMET condition, participants were asked to choose one of the four words that best described the mental state of the person in the black-and-white photograph; in the control condition, the participants were asked to respond to the same facial stimuli and choose the words that accurately described the physical properties of the person, including sex and age.

**FIGURE 1:**
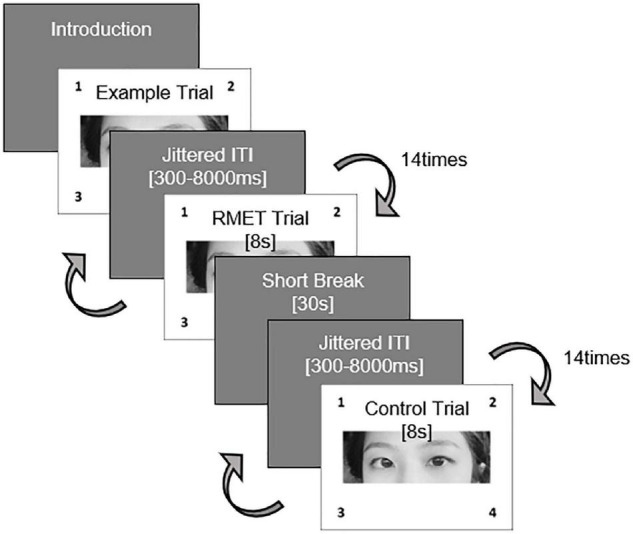
fMRI task design using RMET.

Each condition consisted of 28 trials in a pseudo-randomized order and started with example trials. A total of 56 trials, divided into two sessions, were preceded by one practice trial. Each trial was shown for 8 s, separated by a jittered intertrial interval (35) with a white screen [Inter-trial range varying between 600 and 8,000 ms, average = 3,000 ms]. Participants were asked to respond by pressing a button within 8 s (Figure 1). When the reaction was not completed within the specified time, the participants were provided with a feedback message for 1 s stating “too late,” for 1 s, and the response was subsequently not recorded. To implement the fMRI experiment, we used E-prime 3.0 (Psychology Software Tools) to measure the reaction time (RT) and accuracy of each item.

### Data analyses

#### fMRI data preprocessing

We preprocessed the structural T1-weighted images and EPI functional images using Statistical Parametric Mapping version 12 software (SPM 12^1^). Slice-timing correction was first executed, with the mean images being corrected to compensate for head motion. The EPI images were registered on T1-weighted images. Gray matter, white matter, and cerebrospinal fluid were segmented with each other, serving the forward deformation field. Normalization to an EPI template based on the Montreal Neurological Institute (MNI) reference brain was performed. The images were analyzed using a 6-mm full-width at half-maximum isotropic Gaussian kernel. None of the participants had head movements exceeding 3-mm translation and 3° rotation.

#### fMRI statistical analyses

General linear model (GLM) analyses included six motion parameters (three translation parameters, in millimeters; and three rotation parameters, pitch, roll, yaw; in degrees) defined as square-wave regressors for each stimulus presentation block convolved with a gamma function to approximate the idealized hemodynamic response and one regressor for experiment condition, which are onset durations of the stimuli modeled in regressors of interest only satisfied with the answer.

In the first-level analysis, we compared *RMET minus control (RMET > control)* contrast to obtain an activation map that indicated only emotional identification, based on the GLM using SPM 12. For the second-level analysis, we used a two-sample *t*-test to show the difference in neural activation maps between the groups. In this study, we used a threshold of uncorrected *p* < 0.001 with a cluster size of 20 voxels (*k* ≥ 20) (*T* = 3.45), which is stricter than Lieberman’s proposal (uncorrected *p* < 0.005, *k* ≥ 20 voxel threshold, equivalent to a false discovery rate of 0.05) which was suggested with the aim of minimizing the risk of type I (false-positive) errors (54–56). We also used the significant clusters as a region of interest (57, 58) to analyze the correlation with clinical measurements. ROI activation was extracted using the MarsBaR toolbox^2^, and the extracted values were analyzed using the SPSS^®^ ver 25.0 (IBM Corporation, Armonk, NY, United States) and Microsoft Office Excel software.

#### Non-fMRI statistical analyses

We also conducted a two-sample *t*-test and chi-squared test to analyze participants’ demographic features, self-reported questionnaires, and task results. Correlational analyses (27 NSSIs, 38 controls; one missing data from NSSI was excluded in this test) were also performed to test whether neural activations during RMET performance were related to clinical symptom severity using Pearson’s correlation analysis.

## Results

### Sample characteristics and task performance results

There were no statistical group differences in demographic data (age, sex, education, estimated IQ, and handedness), RT, or accuracy both in the RMET and control conditions (*p* > 0.05 for all). In terms of the clinical measures, individuals with NSSI scored significantly higher on TAS-20 for alexithymia, DERS for emotion dysregulation, and BSI for suicide ideation compared to the controls [*t* (64) = −6.19, *p* < 0.001; *t* (64) = −5.53, *P* < 0.001; *t* (64) = −5.99, *p* < 0.001, respectively]. There was also no group difference in handedness as assessed by the Edinburgh Handedness Inventory (Table 1).

**TABLE 1:** Demographic, clinical, and task performance data of the study participants.

	NSSI (*N* = 28)	Controls (*N* = 38)	Statistics
***Demographic data***			
Age (yrs)	22.96 ± 2.94	22.74 ± 2.72	*t*(64) = −0.25, *p* = 0.80
Sex (M/F)	7/21	12/26	*x*^2^(1) = 0.34, *p* = 0.17
Education (yrs)	14.57 ± 1.97	14.69 ± 1.77	*t*(64) = 0.04, *p* = 0.97
Estimated IQ	109.14 ± 9.26	111.09 ± 9.10	*t*(63) = 0.93, *p* = 0.36
Right handedness (%; n)	89% (25)	84% (32)	*t*(64) = −0.72, *p* = 0.47
***Clinical measures***			
TAS-20	54.48 ± 11.05	39.87 ± 7.99	*t*(63) = −6.19, *p* < 0.001
DERS	75.11 ± 18.12	61.97 ± 11.70	*t*(63) = −5.53, *p* < 0.001
BSI	11.78 ± 6.77	3.53 ± 2.74	*t*(63) = −5.99, *p* < 0.001
***fMRI task performances***			
RMET cond. accuracy (%)	79.53 ± 9.82	79.35 ± 11.47	*t*(64) = 0.48, *p* = 0.63
RMET cond. RT (86)	3.71 ± 0.65	3.60 ± 0.49	*t*(64) = −0.79, *p* = 0.43
Control cond. accuracy (%)	88.87 ± 6.73	89.78 ± 8.11	*t*(64) = −0.07, *p* = 0.95
Control cond. RT (86)	2.68 ± 0.60	2.55 ± 0.51	*t*(64) = −0.96, *p* = 0.34

TAS-20, Korean version of the 20-Item Toronto Alexithymia Scale; DERS, Korean version of the Difficulties in Emotion Regulation Scale; BSI, Korean version of the Beck Scale for Suicide Ideation; RMET cond., fMRI condition using Reading the Mind in the Eyes Test, Control cond., fMRI condition using control task; RT, reaction time.

### fMRI results

#### Brain activation in response to RMET condition

**FIGURE 2:**
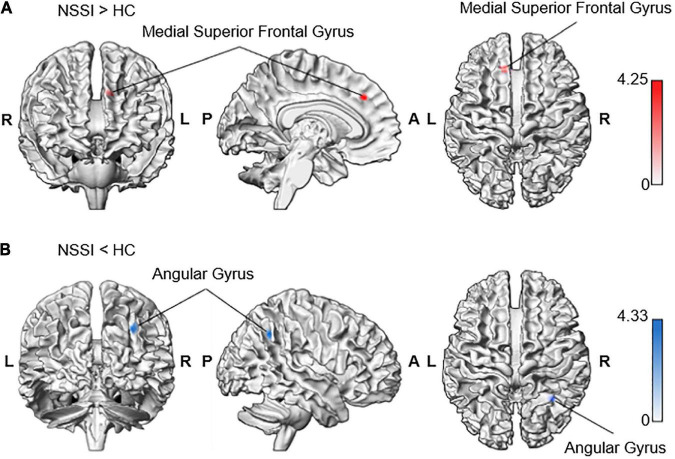
Increased neural response to RMET minus control condition contrasting NSSI and control groups. The whole-brain analysis indicated increased activation during RMET in **(A)** the left medial superior frontal gyrus in the NSSI group (−10, 32, 36; *Z* = 3.98) and **(B)** the right angular gyrus in the control group (32, −54, 40; *Z* = 4.04). Significant clusters were visualized using the xjView toolbox (https://www.alivelearn.net/xjview) with MATLAB.

In the whole-brain analyses, we found that the NSSI group had a significantly increased Blood-oxygen-level-dependent activity in the left superior frontal gyrus and medial part [Brodmann area (BA) 8] [medial superior frontal gyrus; MNI coordinates (*MNI*_*xyz*_) = (−10, 32, 36), *Z* = 3.98, _*uncorrected*_ *P* < 0.001, *k* = 21, peak level *T* = 4.25, *RMET minus control* condition contrast = 0.72, *S*.*E*. = 0.17; interval = 0.56; *beta* = (0.57; −0.15)] during the RMET task (Table 2). In contrast, the NSSI group showed less neuronal activity in the right angular gyrus (BA 39) [AG; *MNI*_*xyz*_ (32, −54, 40), *Z* = 4.04, _*uncorrected*_ *P* < 0.001, *k* = 29, peak level *T* = 4.33, *control minus RMET* condition contrast = 1.00, *S*.*E*. = 0.23; interval = 0.76; *beta* = (−1.42; 0.42)] compared to the controls (Figure 2 and Table 2).

**TABLE 2:** Brain regions exhibiting significant activity differences between NSSI and control groups during RMET task performance.

Condition	Structural region	BA		MNI coordinates			Cluster size	*Z*
				X	Y	Z		
NSSI > HC	Superior frontal gyrus, medial part	8	L	−10	32	36	21	3.98
NSSI < HC	Angular gyrus	39	R	32	−54	40	29	4.04

In response to RMET minus Control condition, there was hyperactivity in the superior frontal gyrus, left medial part (BA 8), and hypoactivity in the angular gyrus, right part (BA 39) in the NSSI group compared to the control group.

#### Relationship between fMRI activation and clinical symptom severity

**FIGURE 3:**
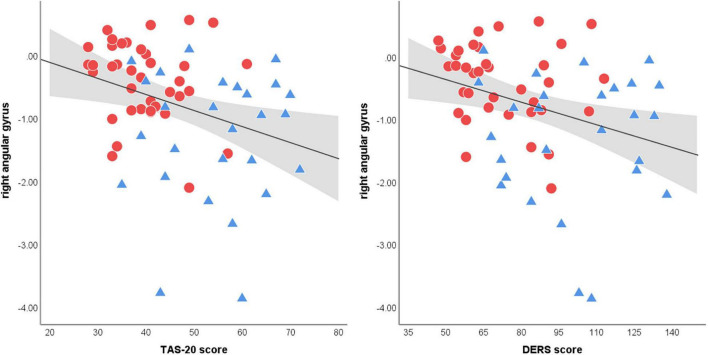
Scatter plots of the correlation between right angular gyrus activation during affective ToM processing and clinical measures. Both Toronto Alexithymia Scale (TAS-20) and Difficulties in Emotion Regulation Scale (55) scores shows significant correlations with angular gyrus activations (spearman’s *r* = −0.37. *p* = 0.002; spearman’s *r* = −0.37. *p* = 0.003, respectively). The linear trend-line is demonstrated in black and the area colored in gray represents 95% confidence intervals of mean scores (red circle = NSSI group, blue triangle = control group).

There was no correlation between the BOLD data during RMET performance and indicators of NSSI severity (i.e., NSSI versatility and NSSI duration) and clinical measures within each group. However, across all participants, brain activation in the right angular gyrus, which appeared to be decreased in the NSSI group compared to the control group, was negatively correlated with the TAS-20 (*r* = −0.37, *p* = 0.002) scores and DERS (*r* = −0.37, *p* = 0.003) scores (Figure 3). There was no correlation between medial superior frontal gyrus responses to the affective ToM task and clinical measures (all *p* > 0.05).

## Discussion

Affective ToM is essential for inferring, responding to, or caring about how the other person feels (59, 60). This socio-affective ability is known to predict an individual’s level of social functioning and quality of life independent of neurocognitive function (61–63). This study aimed to explore the neural correlates of affective ToM in medication-free individuals engaged in NSSI and determine its relationship with the clinical features of NSSI. We evaluated the participants’ affective ToM by examining their performance on the RMET, which is known to capture the nuances of an individual’s affective ToM and reflect socio-affective functioning in real-world situations. In this study, although the behavioral experiment consisting of RMET showed no group differences in accuracy and response time on the neural level, representative areas of the social brain network, which had been lacking in research in the NSSI group (43, 64), revealed disproportionate activation to social-emotional stimuli in this study. That is, the individuals with NSSI showed increased medial prefrontal cortex activation and reduced right angular gyrus activation during the RMET. The main findings from the NSSI group are described in detail below.

### Increased medial superior frontal gyrus activation in NSSI

People with a history of NSSI showed superior accuracy in detecting unfavorable social signals (65). Klonsky et al. (3) has also pointed out a tendency of individuals with NSSI to hypermentalize. According to a biosocial theory of the etiology of self-harm (66), the hypersensitivity to affective stimuli and negative social cues lead to self-harm. Sansone and Sansone (67) have also revealed that interpersonal hypersensitivity can trigger self-harm behaviors. Here, we found increased medial superior frontal gyrus activity in the NSSI group relative to controls during the affective ToM task. The medial superior frontal gyrus plays a key role in affective ToM (31), and increased activation of this area indicates spontaneous mentalization that causes fundamental attribution error (68). A previous fMRI study found hyperactivation of the medial superior frontal gyrus in adults with high sensitivity to the task of discriminating between genuine or posed facial displays (69). In line with this, increased medial superior frontal gyrus activation has also been observed in patients with post-traumatic stress disorder who process negatively valenced information (e.g., fearful faces) (70) and in violent patients with schizophrenia responding to negative emotional pictures and even neutral emotional pictures (71). Given these previous findings, our results of medial superior frontal gyrus hyper-response to socio-affective stimuli suggest neural evidence of hypermentalizing and hypersensitivity to emotional situations in individuals engaging in NSSI. This may involve the risk of emotional dysregulation of NSSI (72, 73).

Conversely, it should be noted that affective ToM function is closely related to the ability of emotion recognition as well (40). Mier et al. (38) also argued for the role of the superior frontal gyrus activation in emotion processing as well as in affective ToM. In view of this, we need to consider the possibility that hyperactivation of this brain region may reflect disrupted neural processing of emotion recognition in NSSI people. Thus, further studies considering the double dissociation approach are needed to evaluate the individual neurobiological underpinnings of emotional recognition and affective ToM in individuals engaging in NSSI.

### Decreased right angular gyrus activation in NSSI

We found that the activation in the right angular gyrus was decreased in individuals with NSSI during the RMET condition. The angular gyrus, the dorsal part of the TPJ, is involved in perspective-taking and ToM (73). In particular, the right angular gyrus is an area showing significant activation in affective ToM conditions (38). More specifically, this area is implicated in self–other distinction and self-inhibition in the existence of conflict between one’s own and others’ perspectives (74–76).

As such, it might be that the attenuated angular gyrus during affective ToM condition signifies a diminished capacity for self–other distinction at the mental-level (77), leading to inappropriate emotional distress (78). In addition, Ramsey, Hansen (76) argued that we automatically compute others’ perspectives even before we explicitly recognize it. Likewise, previous literature has suggested that self–other distinction is a crucial component in accurately understanding others’ mental states (79). Impairment of self–other distinctions or self-perspective inhibition can cause limited and undifferentiated emotional experiences. In line with this, research using a sample of adolescents who reported self-cutting hypothesized that those with NSSI may be hypersensitive to others’ mental states, which may overwhelm their ability to cope with inner experiences (80). In addition, given that the right angular gyrus serves as an inhibitory neural network regulating cognitive, emotional, and motor processes (81), failure to recruit this region in relation to socio-affective stimuli may lead to self-regulation deficits and increased risk of NSSI.

In particular, we found that the decreased magnitude of the neural response in the right angular gyrus was correlated with increased TAS-20 and DERS scores for all participants. Alexithymia and emotional dysregulation are both closely linked to self-injurious behaviors (13, 17, 82). From these results, we speculate that altered responses in the angular gyrus to socio-affective stimuli are potential biomarkers of NSSI risk. However, further detailed research is needed to elucidate the mechanisms by which aberrant neural activity leads to self-injurious behavior. Given the low reliability of the relationship between self-reported measures and voxel-wise neural activity during fMRI tasks, strict replication is required (83).

### Limitations

Several limitations should be considered when interpreting our findings. First, because our study’s cross-sectional design cannot establish causality, so the current findings should be interpreted with caution. In addition, given that emotion recognition and affective ToM are intertwined, and that extensive brain networks are involved in emotion regulation and social information processing (84, 85), region of interest-to-region of interest (ROI-to-ROI) analysis regarding the emotion processing network can also be employed to explore the relationship among brain regions for socio-affective traits of NSSI with greater clarity in the future. Another limitation is the lack of data for confidence in decision-making during RMET, as well as the lack of a classification of the emotional valence of stimuli. Population structure regarding sex ratio in this study can be considered as a limitation as well. Uneven sex distribution in NSSI people is a constant, inevitable problem in NSSI research. Additional studies are needed to confirm the characteristics of emotional processing according to sex using a larger sample size. Finally, no cognitive ToM task was employed as a baseline condition. The development of fMRI tasks to examine biased emotion perception and emotional decision-making may assist in further elucidating the neural correlates in NSSI pathology related to emotion processing.

### Conclusion

Identifying the neurobiological correlates of NSSI is critical for building a pathological model of NSSI. To date, strong neuroimaging evidence has shown a relationship between NSSI and emotion dysregulation; however, the understanding of the neurophysiology related to the socio-affective processes of NSSI remains limited. This study provides the first neuroimaging evidence of a functional imbalance within the social brain network during affective ToM processing in patients with NSSI and its clinical implications regarding alexithymia and emotion dysregulation. In particular, the current neuroimaging studies have postulated that increased medial prefrontal cortex activation and reduced right angular gyrus are promising correlates for the socio-affective processes of NSSI. A better understanding of the pathophysiological mechanisms of NSSI may allow us to offer suggestions for the development of evidence-based treatments or interventions targeting NSSI behavior.

## Data availability statement

The raw data supporting the conclusions of this article are available from the corresponding author on reasonable request.

## Ethics statement

The studies involving human participants were reviewed and approved by Korea University Institutional Review Board. The patients/participants provided their written informed consent to participate in this study.

## Author contributions

GN, HM, and J-WH contributed to the conception and design of the study. GN organized the database. HM performed the statistical analysis and wrote the first draft of the manuscript. All authors contributed to the manuscript revision, read, and approved the submitted version.

## Conflict of interest

The authors declare that the research was conducted in the absence of any commercial or financial relationships that could be construed as a potential conflict of interest.

## Publisher’s note

All claims expressed in this article are solely those of the authors and do not necessarily represent those of their affiliated organizations, or those of the publisher, the editors and the reviewers. Any product that may be evaluated in this article, or claim that may be made by its manufacturer, is not guaranteed or endorsed by the publisher.

## Footnotes

1
http://www.fil.ion.ucl.ac.uk/spm/

2
http://marsbar.sourceforge.net/
